# Author's response to: "Harmful effects form one puff of shisha-pen vapor: methodological and interpretational problems in the risk assessment analysis"

**DOI:** 10.1186/s12971-016-0087-6

**Published:** 2016-07-07

**Authors:** Peter M. J. Bos, Anne S. Kienhuis, Reinskje Talhout

**Affiliations:** National Institute for Public Health and the Environment, Bilthoven, The Netherlands

We thank Dr. Farsalinos and Dr. Baeyens for their comments in their letter to the editor on our publication: “*Potential harmful health effects of inhaling nicotine*-*free shisha*-*pen vapor*: *a chemical risk assessment of the main components propylene glycol and glycerol*” [1]. Apparently some issues discussed in our publication were not sufficiently clear and we thank the editor of *Tobacco Induced Diseases* for the opportunity to respond to these comments and clarify the issues raised.

The comments made by Dr. Farsalinos and Dr. Baeyens focus on four specific issues. Their main concern however, is about the applicability of our proposal to use the Margin of Exposure (MOE) approach for risk assessment of exposure via shisha-pens or electronic cigarettes (e-cigarettes). Therefore, we would like to elaborate first on the application of this approach as a method of risk assessment for exposure to chemicals via smoking and vaping and why we are of the opinion that, at present, this is the best method for the present exposure scenario.

The exposure pattern in smoking or vaping is rather complex. E-cigarettes or shisha-pens may be used during one or more vaping sessions per day and a session consists of a variable number of puffs. Thus during a day, exposure is intermittent in two ways, i.e., time intervals exist between sessions and between puffs within each session. Since exposure occurs only when a puff is taken, the daily exposure consists of multiple peak exposures with irregular time intervals in between. The exposure assessment is further complicated by the large variation in the use of e-cigarettes. Based on a market survey of the use of e-cigarettes in The Netherlands, it has been estimated that the number of puffs during a day may be up to 500 (or even larger in individual cases) with a total duration (i.e., total sum of session duration) of 4 h per day. The question then raised is how risks can be estimated from such a complex exposure scenario. The available human limit values for respiratory exposures are not suitable. Occupational exposure limits (OELs) are not applicable to the general population and are generally meant for 8-h exposures while limit values for the general population (such as Air Quality Guidelines as published by the WHO) are generally derived for continuous exposure, i.e., 24 h per day for 7 days per week. The exposure scenarios for which these limit values are derived do not match the exposure pattern for vaping.

Hence, the question then is how to proceed if one wants to assess the health risks from the use of e-cigarettes or shisha-pens? We believe that the best option forward is the application of the Margin of Exposure (MOE) analysis. This approach is commonly used within different risk assessment frameworks and has been used before in a risk assessment for exposure to 1,3-butadiene through smoking [2]. The MOE approach has also been proposed in risk assessment of smoking by others (e.g., [3, 4]). A manuscript describing the application of the MOE within risk assessment of smoking or vaping in more detail is in preparation.

An MOE is the ratio of a reference point (the PoD), often taken from an animal study and corresponding to an exposure that causes a low but measurable response, and the exposure estimate in humans, taking into account differences in vaping behaviour (adapted from [5]). The MOE approach starts with the choice of an appropriate PoD that is derived from an exposure scenario that closely resembles the exposure scenario under evaluation and preferably is based on human data.

In general, only interspecies and inter-individual differences in susceptibility need to be taken into account if no adverse effects are observed at the PoD, provided that the exposure pattern from which the PoD is derived sufficiently resembles the human exposure scenario under evaluation. Typically, an MOE of minimally a factor of 100 is then required (i.e., a factor of 10 each for interspecies and intraspecies differences) to reach a conclusion of no concern, i.e., that no adverse health effects are to be expected. In case a PoD is derived from adequate human data, interspecies differences do not need to be accounted for and an MOE of 10 might be concluded sufficient.

If the exposure scenario on which the PoD is based differs from the regarding human exposure scenario, these differences need to be bridged by taking them into account in the evaluation whether an MOE is sufficient to reach a conclusion of no concern. For instance, if the PoD is derived from a 13-week animal study a larger MOE is warranted if human exposure is lifelong. In contrast, a lower MOE may be considered sufficient if human exposure is for instance only for one hour per day whereas the PoD is based on a 6-hour exposure per day. By considering all factors involved in the risk assessment of a human exposure scenario a minimum value for the MOE may be determined that is required to reach the conclusion that no adverse health effects are to be expected.

As to the human exposure scenario of vaping however, the exposure scenario is so complex that it is difficult to clearly quantify the differences between exposure through vaping and for instance a 6-hour exposure in an animal experiment. This hampers the assessment of a clear minimum value required for the MOE. Animal experiments with an exposure pattern that mimics the exposure during the use of an e-cigarette are not available. Furthermore, the question would arise what the appropriate exposure pattern in the animal experiment would be, considering the large inter-individual variation of e-cigarette use. The more intensive the vaping behaviour, the higher the MOE should be to reach a conclusion of no concern. The best way forward then is to calculate the MOE, to list the factors to be accounted for by the MOE, to quantify these factors where possible and to include the remaining not-quantifiable factors in the evaluation of the MOE by expert judgement. It is noted that the minimal value required for the MOE depends on the information available and on the exposure characteristics and thus will be different for different scenarios.

In general, the interval in between two puffs will be sufficiently long such that the alveolar concentration will have decreased to zero before the next puff. Therefore, as a first approach the exposure from a single puff can be estimated and compared with the most appropriate PoD available. The exposure assessment may either be an estimate of the pulmonary or alveolar concentration (if local effects are the endpoint of concern) or of the absorbed dose (in case systemic effects are of interest). The exposure estimate for a single puff can then easily be extrapolated to more intensive users. In case of local effects, such as respiratory tract irritation, the total duration of the daily exposure can be estimated from the total duration of the daily vaping sessions combined with the puff frequency during these sessions. Combined with the exposure concentration estimated for a single puff this provides an estimate of the daily exposure that can be used for the calculation of the MOE. Any remaining differences can be considered in the evaluation of the MOE, including the fact that exposure is actually not continuous but to peaks with intermediate zero alveolar concentrations. As to systemic exposure, the dose taken up from one puff can easily be multiplied by the total daily number of puffs to estimate the total daily systemic dose. However, it should then be kept in mind that the rate at which a substance enters the systemic circulation is also of importance. This aspect might be taken into account by additionally including the puff frequency in the evaluation.

In summary, the risk assessment of the use of e-cigarettes or shisha-pens is complex but the MOE approach can be used to obtain a first pragmatic risk estimate, and to our opinion is at present the best option despite the uncertainties involved. The exposure from a single puff can be estimated and extrapolated towards more intensive use, depending on whether one wants to assess the health risks for e.g., a light, mediate or heavy user of e-cigarettes or, as in the present case, of shisha-pens. Therefore, the focus of our paper was on single-puff exposure that could easily be extrapolated to another vaping behaviour of choice rather than focusing on possible risks in one specific well-described vaping scenario. Apparently, our paper was not very clear on this point. Obviously, a single-puff exposure will be insufficient to induce adverse health effects.

Having said that, we come back to the four specific issues raised by Dr. Farsalinos and Dr. Baeyens. The first point raised regards the risk assessment for propylene glycol. Although the purity of the propylene glycol used by Wieslander et al. [6] is indeed unknown even industrial grade propylene glycol may have a purity of more than 99.5 %^1^. Further, it is well-known that propylene glycol has irritating properties, due to its dehydrogenating properties [7]. It can therefore be safely assumed that the findings reported by Wieslander et al. [6] are induced by propylene glycol and that any impurities present in the aerosol mist, will have had a minor contribution, if any.

We estimated that the alveolar concentration of propylene glycol after one puff may be as high as approximately 500 mg/m^3^, comparable to the concentrations reported by Wieslander et al. [6] that induced upper airway irritation. The alveolar concentration is also significantly higher than the lowest concentration of 160 mg/m^3^ tested in a 13-week rat study, which was an effect level for nasal haemorrhage [8]. In case of a lifetime human exposure scenario of 6 h per day, the minimum value required for an MOE would be at least about 200 (i.e., 10 for interspecies, 10 for interindividual differences in susceptibility, 2 for subchronic to lifetime extrapolation), meaning that a safe exposure concentration for a daily 6-h exposure for humans would be less than 1 mg/m^3^. To which extent a shorter daily exposure duration then balances a much higher exposure concentration cannot be verified due to insufficient data. However, the estimated alveolar concentration after one puff in itself is sufficiently high to potentially induce adverse effects on the respiratory tract. The effects reported both by Wieslander et al. [6] and by Suber et al. [8] are clearly unwanted from a risk perspective point of view and are therefore considered as adverse. It can therefore be safely concluded that the heavier the daily use of a shisha-pen the higher the possibility that adverse health effects on the upper airways will occur, although the minimum number of daily puffs at which effects can be expected cannot be assessed due to insufficient data. This minimum number will also differ between individuals, since individuals show differences in susceptibility towards hazardous chemicals.

Furthermore, in the light of the present discussion it is interesting that at the FAQ website of *The members of the Propylene Oxide*/*Propylene Glycol sector group of CEFIC*^2^ one of the questions is whether the use of propylene glycol in e-cigarettes is safe. The answer states that “*The producers of propylene glycol and members of Cefic*’*s PO*/*PG sector group do not support the use of propylene glycol in electronic cigarettes*, *nor in artificial* (*theatrical*) *fogs due to possible effects on the eye*, *nose*, *throat*, *and respiratory tract membranes as well as the absence of information on potential long term effects from prolonged inhalation of* (*fine*) *droplets of propylene glycol*.”

In summary, the main point that we would like to stress is that in principle the alveolar concentration following one puff is sufficiently high to potentially induce adverse effects on the upper respiratory tract. Whether these effects will occur obviously depends on the number of puffs, the puff frequency and/or on the total duration of daily exposure. It cannot be ruled out that even brief peak exposures to such high concentrations may induce adverse effects provided that the number of peaks is sufficiently high. The effects reported by Wieslander et al. [6] in humans exposed for only one minute might indicate that for propylene glycol the use of shisha-pens might not need to be much more intensive before adverse effects can be expected. The fact that Dr. Farsalinos and Dr. Baeyens state that “throat irritation is commonly called “throat hit” by smokers and vapers” indicates that these effects are known among vapers. This is further underpinned by their remark that complaints including dry/irritated mouth and throat occurred among e-cigarette users.

The same issues as discussed for propylene glycol also hold for the risk assessment for glycerol, which is the second point raised. Also for glycerol, the estimated alveolar concentration is high (approximately 400 mg/m^3^) and of the same order of magnitude as the concentrations that induced adverse effects on the respiratory tract in rats exposed for 6 h/day for 13 weeks [9]. As mentioned before in the response but also already in our paper [1], we acknowledge that a 6-h daily exposure significantly differs from the brief peak exposures following each puff from a shisha-pen. But, similarly as for propylene glycol, the heavier the use of a shisha-pen the higher the possibility for adverse effects on the respiratory tract due to glycerol exposure.

As to the third point, we agree with Dr. Farsalinos and Dr. Baeyens that the statement in our manuscript that the effects reported by Suber et al. [8] are irreversible, is inaccurate. Although no recovery group was included in the study it can be fairly concluded that the effects reported (including the nasal haemorrhages, the elevation in the number of goblet cells and the elevated mucin production) will be reversible. However, we would like to emphasize that whether an effect is reversible or not is only of importance in case of cessation of exposure. In case of lifelong exposure the fact that an effect is (ir)reversible does not alter the conclusions of a risk assessment itself.

As a last point, Dr. Farsalinos and Dr. Baeyens plead that the evaluation of the use of e-cigarettes should always be in the context of smoking since e-cigarettes are mainly used as an alternative to smoking. However, e-cigarette use is subject of discussion of opposite camps and we believe the discussions are best helped forward if potential health risks of e-cigarette use are judged on their own merits. As to the shisha-pen, which was the main focus of our paper, there was more reason to discuss possible risks from shisha-pen use in itself, since the marketing of shisha-pens also aims at children or teen-agers.

We approached the use of shisha-pens from the perspective of risk assessment, irrespective of the experiences and opinions of vapers. This includes that effects, causing a diminished functioning of a tissue or organ or a decreased well-being, are considered as unwanted and therefore adverse. Within that context irritation of the respiratory tract is considered to be an adverse health effect. We believe it is the task of a risk assessor to clearly point out health risks involved in exposure to hazardous chemicals, thereby including actions leading to such an exposure. If a person is willing to take such a risk it is an individual’s choice.

Considering the increasing use of e-cigarettes and the lack of insight in the actual risks involved in their use, it is of importance to develop a method to assess these risks. Applicable human limit values are not available. Therefore, we believe that the MOE approach is the best option forward. However, at the same time we acknowledge that the data that are actually needed for this approach are not available yet. This holds both for an appropriate exposure assessment as for the necessary toxicological information needed to address the exposure scenario of smoking or vaping. The MOE can be used as a first pragmatic approach and by application it will reveal the uncertainties involved and thereby make clear what kind of data is needed to adequately address human risk assessment from smoking or vaping. We hope that future research on e-cigarettes will also focus on filling in data gaps to improve the assessment of health risks from vaping and/or smoking.

## Footnotes

1 http://msdssearch.dow.com/PublishedLiteratureDOWCOM/dh_0935/0901b8038093558e.pdf?filepath=propyleneglycol/pdfs/noreg/117-17101.pdf&fromPage=GetDoc

2 http://www.propylene-glycol.com/faq
